# A Critical Review on Communication Mechanism within Plant-Endophytic Fungi Interactions to Cope with Biotic and Abiotic Stresses

**DOI:** 10.3390/jof7090719

**Published:** 2021-09-01

**Authors:** Hongyun Lu, Tianyu Wei, Hanghang Lou, Xiaoli Shu, Qihe Chen

**Affiliations:** 1 Department of Food Science and Nutrition, Zhejiang University, Hangzhou 310058, China; luhongyun@zju.edu.cn (H.L.); 21913067@zju.edu.cn (T.W.); louhanghang@zju.edu.cn (H.L.); 2 College of Agriculture and Biotechnology, Zhejiang University, Hangzhou 310058, China; shuxl@zju.edu.cn

**Keywords:** to-cell communication, plant-microbe interactions, common symbiosis signaling pathway, endophytic fungi

## Abstract

Endophytic fungi infect plant tissues by evading the immune response, potentially stimulating stress-tolerant plant growth. The plant selectively allows microbial colonization to carve endophyte structures through phenotypic genes and metabolic signals. Correspondingly, fungi develop various adaptations through symbiotic signal transduction to thrive in mycorrhiza. Over the past decade, the regulatory mechanism of plant-endophyte interaction has been uncovered. Currently, great progress has been made on plant endosphere, especially in endophytic fungi. Here, we systematically summarize the current understanding of endophytic fungi colonization, molecular recognition signal pathways, and immune evasion mechanisms to clarify the transboundary communication that allows endophytic fungi colonization and homeostatic phytobiome. In this work, we focus on immune signaling and recognition mechanisms, summarizing current research progress in plant-endophyte communication that converge to improve our understanding of endophytic fungi.

## 1. Current Knowledge of Endophytic Fungi

Plants, as sessile organisms, have evolved in the context of a microbial world [1,2]. The earliest records of plant-microbe interactions date back 407 million years [3]. In spite of a small host-mediated changes have happened in microbiomes, host-associated microbiome changes have significant effects on plant health. Plants are inhabited internally by a multitude of fungi, which can transcend the endodermis barrier free from plant immune signal attack, crossing from the root cortex to the vascular system, and, subsequently, thriving as endophytes in plant organs [4,5]. Genome drafts in plants revealed a large overlap of genome-encoded functional capabilities between leaf- and root-derived bacteria, with few significant differences at the level of individual functional categories [6]. The endophytic fungal community is affected by organelles, plant exudates [7], age, climate [8], nutrient balance, geographical location [9], and season [10]. Host plants’ evolutionary relatedness is also strongly associated with endosphere (microbes within plants) diversity, indicating that hosts’ underlying endosphere assemblies covary with phylogenetic relatedness among hosts [11]. Beneficial endophytes could contribute to the mobilization of nutrients from complex organic matter to the host plant, thus promoting plant growth [12,13]. Moreover, complex networks of mycorrhizal hyphae connect root systems of individual plants, regulating nutrient flow and competitive interaction between and within plant species, controlling seedling establishment, and, ultimately, influencing all aspects of plant community ecology and coexistence [14]. Interestingly, human gut microbiomes have the same function with plant-associated endophytes in host protection, such as absorbing nutrients, defending against pathogen colonization, regulating the host immune response, and distinguishing between friends [15]. Generally speaking, endophytic fungi play an important role in ecological aspects and can be plant biostimulants as well as biofertilizers and biopesticides, offering a promising alternative to ensure sustainability in agriculture without the harmful side effects of chemical fertilizers [16]. Hence, endophytic fungi, nowadays, attract more attention. This review explores our knowledge of communication signaling between endophytic fungi and plants, with the aim of highlighting emerging frontiers in plant-endophyte interaction.

## 2. Access of Fungi into Plant Tissues

Fungi can enter plant tissues through different pathways, including tissue wounds, stomata, lenticels, root cracks, and germinating radicles. Unlike bacterial proliferation, the route of hyphal invasion in plant cell is predicted by a prepenetration apparatus. Arbuscular mycorrhizal fungi (AMFs) are accommodated in root cortical cells, forming arbuscules [17]. The plant responds to mycorrhiza by initiating the common symbiosis pathway (CSP), altering gene expression and root morphology [18]. Moreover, plant shoot tips in nodulated dicot species contain many modified secretory trichomes that arise from the lower epidermis, suggesting an important role of trichomes in host-endophyte interactions [19].

In order to colonize plant tissues, endophytic fungi need to break through the physical barrier of plant cell walls. At the point of contact with a microbe, the plant cell wall is modified and becomes less rigid, allowing plasma membrane invaginations and uptake of the microbe. Consequently, endophytic fungi remain enclosed in membrane compartments and are not in direct contact with plant cytoplasm [20]. The molecular exchange between endophytes and plant cytoplasm takes place through their plasma membrane and cell wall, thus defining a functional compartment called the symbiotic interface. The cell wall is deeply involved in the molecular dialogue between plants and microorganisms, mediating most plant-microbe interactions [21].

Endophytic fungi can degrade the plant cell wall and change its structure by secreting cell wall degradative enzymes (CWDEs) such as cellulase, laccase, pectinase, and xylanase, thereby infiltrating, colonizing and proliferating tissues in plant [22]. The findings of Fourier transform infrared spectroscopy (FTIR) detection demonstrated that pathogens had a similar reaction to the plant cell wall. However, most endophytes do not break down lignin and carbohydrates, resulting in nonpathogenic and asymptomatic responses to infected hosts [22]. Additionally, under the same conditions, endophytes have a stronger ability to infect the host than do pathogens [23]. With the help of comparative genomics and transcriptomics, it is reported that, in addition to honeysuckle hosts, ericoid mycorrhiza (ERM), which often appears in plant species as root endophytes, is similar to saprophyte in its degrading enzyme gene content library of bacteria and pathogens, such as polysaccharide degrading enzymes, lipases, proteases, and some enzymes involved in secondary metabolism [13]. ERM genomes contain a significantly greater number of CAZymes, iron reductases, and quinone-dependent oxidoreductases than ectomycorrhizal (ECM) and rot fungi genomes. Other than ECM genomes with an extensive loss of genes coding for plant CWDEs [24], ERM genomes encode a number of genes coding for lignocellulose oxidoreductases, such as laccases, cellobiose dehydrogenases, along with lytic polysaccharide monooxygenases involved in the cleavage of chitin, cellulose, hemicellulose, and pectin, finally resulting in the difference in the ability of ECM and ERM to infect the host plant. Once the fungus overcomes the epidermal layer, it grows inter and intracellularly along the root in order to spread fungal structures [21].

## 3. Plant Innate Immune System

**Figure 1:**
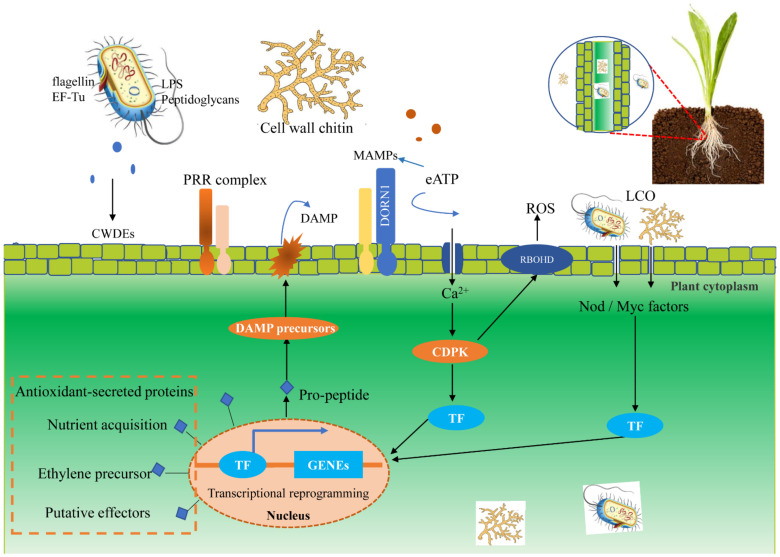
Plant immune recognition of microorganisms and colonization of endophytes in plant cells. Once invaded by microorganisms, the plant can recognize their individual receptors, such as fungal cell wall chitin, and finally make an immune response. On the MAMPs/DAMPs perception of inducing the formation of receptor complexes, PRR activate intracellular signal transduction, which involves Ca^2+^ signaling, CDPK, in a wide range of transcriptional reprogramming intermediate and defense-related TF. In addition, microbial apoplastic eATP can activate the Ca^2+^ signal. The transcriptional expression of plant nuclei can be affected by the colonization of microbe in plant tissues, which may be beneficial for plant survival and endophyte colonization. Abbreviations: microbial/damage-associated molecular patterns (MAMPs/DAMPs), pattern recognition receptors (PRR), Ca^2+^-dependent protein kinase (CDPK), lipo-chitooligosaccharide (LCO), lipopolysaccharides (LPS).

For nutrient acquisition, both beneficial microbe and pathogen can penetrate their host without breaking the plasma membrane of the plant cell. Plants have a genetically imprinted innate immune system that responds to microbe- or pathogen-associated molecular patterns (MAMPs or PAMPs) and pathogen effectors [25]. Plant pathogens can secrete a diversity of virulence proteins and metabolites called effectors, which have evidently evolved to favor pathogen infection. Nevertheless, a subset of them inadvertently activates plant immune receptors [26]. Extracellular recognition of MAMPs or PAMPs and damage-associated molecular patterns (DAMPs) leads to the first layer of inducible defenses, termed pattern-triggered immunity (PTI) [27,28], which plays an important role in preventing nonadapted microbes from infecting the host and in restricting infection of adapted pathogens in susceptible hosts [27,28]. To detect microbe- and host-derived molecular patterns in the first layer defense, plants deploy a large number of receptor-like kinases (RLKs) and receptor-like proteins (RLPs) as pattern recognition receptors (PRRs) [28] (showed in Figure 1). Subsequently, initiating the immune response by PRRs is designated as MAMP-triggered immunity (MTI). Another PTI can be activated by endogenous signals generated upon cellular disintegration, termed DAMPs. DAMPs also are detected by PRRs, playing a key role in innate immunity endogenous elicitors or nonadapted microbes [29]. The degradation compounds of plant cell walls, such as oligogalacturonides and cellodextrins, as well as molecules arising from necrotic, damaged or stressed cells, e.g., cutin monomers and small peptides, can also act as plant DAMPs signals [30]. Additionally, upon attack, effector kinase botrytis-induced kinase1 (BIK1) of the PRRs complex can be activated, triggering an increase in CNGC-mediated cytosolic calcium (Ca^2+^), which is an essential signal for pathogen-associated molecular patterns (PAMPs)-triggered immunity during PTI in plants [31]. In turn, pathogens employ a wide array of virulence effectors to overcome PTI and establish successful infection, termed effector-triggered susceptibility (ETS). In this case, plants will activate a second immune system, displaying an amplified and robust form of defense programs that are termed effector-triggered immunity (ETI), primarily inside the plant cell. Therefore, PTI and ETI can induce plant defense against invading microorganisms by changing the ion flux across the plasma membrane, increasing cytosolic Ca^2+^ and apoplast ROS levels, activating mitogen-activated protein kinase (MAPK), and accumulating nitric oxide (NO), followed by the formation of phytohormones, such as ethylene (ET), jasmonic acid (JA) and salicylic acid (SA), stomata closure, callose deposition, and defense-related transcriptional and metabolic reprogramming [27,32,33]. Therefore, the first line of immune defense derived from plant cells is capable of distinguishing pathogens from endophytes in a MAMP-mediated screening. Additionally, plant pattern-triggered immune signaling, such as MIN7 and CAD1, found in major land plant lineages, is probably a key component of a genetic network through which terrestrial plants control the level and nurture the diversity of endophytic microbiota for survival and health in a microorganism-rich environment [34]. Apart from the above pathways, plant extracellular vesicles (EVs) are lipid compartments capable of trafficking proteins and signaling lipids, RNA, and metabolites between cells, appearing to act as key mediators of plant-microbe interactions, and displaying antipathogen activity in stress responses. During immune responses, plant cells secrete EVs where host-derived small interfering RNAs and microRNAs can silence fungal genes and stress responses, suggesting that plant EVs may also mediate transkingdom RNA interference [35]. Moreover, plants can excrete plant hormones [36] as well as secondary metabolites-containing root exudates, including alkaloids, flavonoids, organic acids, amino acids, and triterpenes, which act as signaling molecules, attractants, and stimulants, but also as inhibitors or repellents to potentially combat pathogen infections and suppress herbivore performance [7,37,38].

## 4. Communication of Endophyte with Plant Tissues

**Figure 2:**
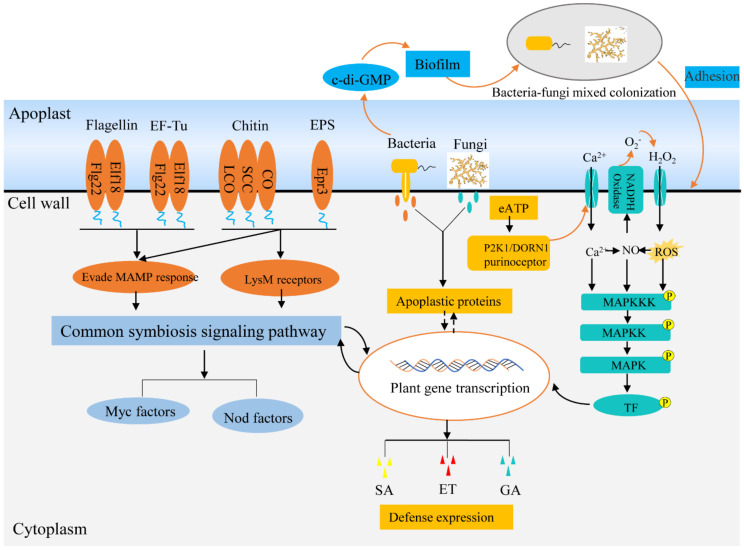
Symbiotic signaling pathways of endophytes within plants. Beneficial endophytes can evade PRR recognition by evolving divergent MAMPs and modifying chitin and polysaccharide components of cell walls, thus suppressing plant immunity to endophyte. Moreover, endophytes can interfere with the company host immune signaling components by secreting effectors, such as apoplastic protein effector, eATP. In addition, endophytes can strengthen the immune response of plants to pathogens and make themselves dominant in plant tissues. Abbreviations: lipo-chitooligosaccharide (LCO), short-chain chitin (SCC), chitooligosaccharide (CO), exopolysaccharides (EPS), nodulation factors (Nod factors), mycorrhizal factors (Myc factors).

Communication among kingdoms is integral to the interaction between plants and endophytes, which is beneficial for establishing a homeostatic phytobiome. However, with the exception of some physical properties (such as light), most communication signals are chemical molecules in nature, including lipids, peptides, polysaccharides, and volatile metabolites. In the plant-microbe interaction, the recognition receptors of endophytes, such as cell wall components and apoplastic proteins, eATP, can produce symbiotic signals with plants. In addition, endophytes have evolved to escape immune signals from plants and thus enter tissues to become symbiotic within plants. Moreover, biofilm formation is more conducive to the adhesion of endophytes to plant tissues and the enhancement of communication between endophytes. Further, colonized endophytes could inhibit plant pathogens. Therefore, the symbiotic signals of endophytes and their tolerance to immune regulation of immune plants and their resistance to the growth competition of pathogens enable them to adapt and maintain homeostasis in plants (summarized in Figure 2).

### 4.1. Microbial Receptors Act as “Identification Code”

When endophytes and pathogens invade plants, they can stimulate the second layer of plant immunity MTI. Though the main function of plants is restricting the microbial load, plants seem to be unable to discriminate between pathogenic and mutualistic microbes [25]. There is a high degree of overlap between symbiotic and immune signaling by exploiting cross-regulations within host PRR pathways. However, plant immune and symbiotic receptors are similar but litter distinct, which trigger entirely different responses to beneficial microbes and pathogens [17]. The recognition of fungal and oomycete pathogenic species by plants is dominated by the perception of microbial components of the cell wall. Lipo-chitooligosaccharide (LCO) signaling derived from microbial cell wall and exudates, also known as Myc factors, as well as short-chain chitin oligomers generated by AM fungi, can be perceived by lysin-motif (LysM) receptors, which are involved in the activation of a common symbiosis signaling pathway (CSSP) [39,40]. Conversely, CSSP, shared by all host plants that establish endosymbioses, has been lost by ECM during long period evolution [41,42]. The main components of fungal cell walls in fungi and β-glucans in oomycetes are immune and symbiotic receptors [17]. Microbial surface polysaccharides, including capsular polysaccharides, lipopolysaccharides, cyclic glucans, and exopolysaccharides (EPS), are important signals for microbial interactions within plants. Additionally, microbe-to-plant signal compounds play a key role in the response to plants. For example, volatile organic compounds (VOCs), which target key points in plant physiology, activating downstream metabolic pathways by a domino effect, are exploited as a source of MAMPs in plant–microbe communication [43,44].

### 4.2. Endophyte-Secreted Apoplastic Proteins and Nucleotides Promote Colonization

Additionally, apoplastic communication produced by plant-associated endophyte have crucial functions in mediating microbial accommodation to defend plant immune signals. Effector-like small, secreted proteins can be used by endophytes to alter the physiological status of the plant host, leading to favored endosymbiosis [45,46]. Apoplastic proteins have major roles in the plant cell wall structure, stress responses, primary and secondary metabolisms, defense, and signaling [47,48]. To evade the first layer of plant MTI defense, colonized microbes can secrete effector proteins, using a conventional secretion system—namely, the golgi-endoplasmic reticulum pathway—into the apoplast where they interact with their molecular targets or are translocated into plant cell cytoplasm to block downstream signals. By using proteomic tools to identify the complex physiological processes among *Trichoderma virens*-maize interactions, it has been demonstrated that apoplastic proteins secreted by the biocontrol fungus are capable of playing crucial roles in plant immune suppression [49]. Moreover, the proteins secreted from *T. virens* are mainly correlated with cell wall hydrolysis, scavenging of ROS, and secondary metabolism, along with putative effector-like proteins. In addition, proteins involved in phytohormones signaling, such as the synthetic precursor protein of ethylene, and cell wall modification, such as glycosyl hydrolases (GHs) proteins, nutrient acquisition, antioxidant secreted proteins, and signal transduction are all identified. The significantly different expression of secreted proteins is determined by the plant [46]. Moreover, extracellular bioactive nucleotides, such as extracellular ATP (eATP), which can induce Ca^2+^ response, MAPK activation, and immune gene expression, as well as its perception DORN1, both play an important role in plant for growth and biotic stress resistance [50]. Additionally, ecto-5′-nucleotidase (E5′ NT), belonging to effector proteins in the apoplastic fluid, can be excreted in plant-microbe colonization rather than axenic culture to counteract eATP release for endophyte living. During colonization by beneficial filamentous root endophyte *Serendipita indica*, eATP accumulates in the apoplast at early symbiotic stages. Additionally, fungal-derived enzymes E5′ NT can modify plant-derived apoplastic nucleotide levels to collectively promote fungal accommodation in plants [49].

### 4.3. Endophyte-Mediated Escape from Plant Immune MAMP-Triggered Response

Commensal and beneficial plant-associated microbes have evolved the ability to evade MAMP recognition directly through suppressing or avoiding MAMP-triggered responses from the host’s defense system, leading to the establishment of a mutual interaction with the host. The plant-microbe interactions can trigger the recognition of cell-cell signaling molecules, such as the chitin component of cell wall recognized by plant PRRs. Nevertheless, endophytes colonized within plants have evolved several strategies to evade or downregulate plant-triggered immune responses. One strategy relies on the secretion of effector proteins that interfere with plant chitin-triggered immunity. Secreted LysM effector identified from the model AM fungal species subvert chitin-triggered immunity in symbiosis [51]. Moreover, to escape early plant defense responses, endophytes can widely change the gene transcript reprogramming of the plant hormone defense signal, which is proceeded by a transient repression of plant immune responses, supposedly to allow root colonization [52]. It has been reported that, after incubation with endophytic fungus for 24 h, most of the detected *Arabidopsis* defense-related genes, mediated by JA and SA, have been downregulated [53]. Local suppression of root immune responses is a common feature of ISR-eliciting beneficial microbes, which possibly aids in root colonization [54]. Notably, during adaptation to plant environment, endophytes can produce a co-occurrence of plant-associated gene clusters and plant-shared protein domains, via binding to extracellular microbial mannose molecules and acting as a molecular invisibility cloak [55,56]. Correspondingly, proteins produced by endophytes serve as molecular mimics to interfere with plant immune functions through the disruption of key plant protein interactions and represent a strategy of avoiding immunity triggered by MAMP. It is demonstrated that MAMP-repressed genes in endophytes have a strong auxin signature [57], which can be beneficial for finely balancing growth-promoting and defense-eliciting activities of beneficial microbes in plant roots by dual role for auxin signaling. As for the indirect method of evading immune signals, *FGB1* and *WSC3* genes derived from a plant root endophyte, such as *Piriformospora indica,* can encode fungal-specific β-glucan-binding lectin, a kind of MAMP, to efficiently suppress β-glucan-triggered immunity in different plant hosts by altering fungal cell wall composition and properties [58,59]. Apart from the above pathways, some growth-promoting and ISR-inducing endophytes have evolved a good tolerance for antimicrobial agents, such as coumarin scopoletin released in response to plant immunity, resulting in plants selectively inhibiting soil-borne fungal pathogens, without affecting the endophyte [60]. Moreover, plant roots can also request cell damage to mount a strong and localized immune response caused by high amounts of beneficial microbes as well as pathogenic, damage-inducing bacteria [61]. With the strong colonization of beneficial endophytes within seedling roots, insignificant MAMP responses are observed in undamaged, differentiated roots. However, when cell ablation is combined with colonization, some neighboring plant cells can show a MAMP response to endophytes. Hence, although endophytes that evade MTI surveillance in plants can still activate the MAMP signal from the plant cell immune system, conversely, root pathogen colonization initially does not cause cell damage, nor a strong MAMP response. However, infection progression eventually leads to the cell death of some epidermal cells, associated with a localized upregulation of MAMP responses in neighboring cells. Damage-gating of the plant root can minimize immune responses against nonpathogenic root colonizers with a tolerant attitude. With pathogens, due to their innate destructive effects, plants will immediately recognize damaged cells and efficiently turn on the “switch” of immune response with a “zero tolerance attitude” to prevent further invasion of local pathogens [61].

### 4.4. Endophytic Fungi Defense against Pathogens through Plant Immune Responses

**Figure 3:**
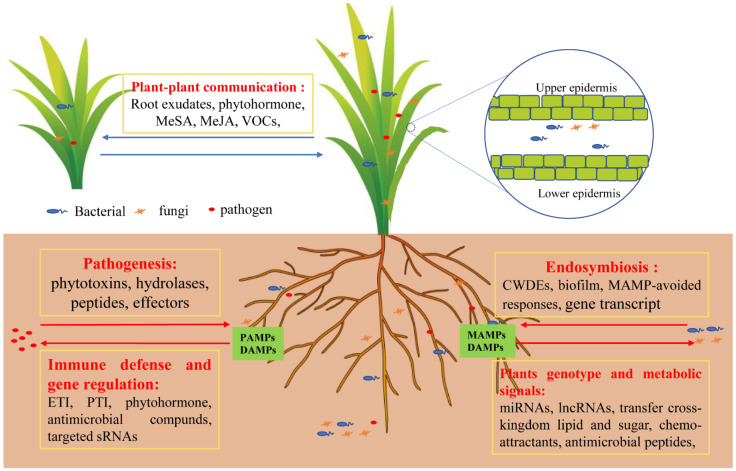
Immunity and symbiotic regulation of plants to microorganisms. Pathogens secrete phytotoxins, hydrolases, peptides, and other effectors. Plants activate the immune defense signaling response, such as ETI and PTI, and secrete phytohormones, antimicrobial compounds, or target sRNAs via gene regulation. Certain types of bacteria and fungi termed endophytes are allowed to enter plant cells by secreting biofilm or by using hyphae to attach to plant tissues and secrete CWDEs to degrade plant cell walls. Moreover, it can regulate gene transcription through evolution to escape plant immune signals and produce symbiotic signals as well as collectively resist pathogen attacks. Accordingly, plants secrete chemo-attractants to attract beneficial endosymbiotic microbes, evolve their own genotype, and transfer cross-kingdom lipid and sugar to endophytes, providing a good parasitic environment for endophytes. Hence, a plant’s genotype can influence the microbiome composition and shape their microbiome to enhance defense and mitigate the trade-off between growth and defense against pathogens. Abbreviations: cell wall degradative enzymes (CWDEs), methylsalicylic acid (MeSA), methyljasmonic acid (MeJA), ectomycorrhizae (ECM), microbe- or pathogen-associated molecular patterns (MAMPs or PAMPs), damage-associated molecular patterns (DAMPs), pattern-triggered immunity (PTI), effector-triggered immunity (ETI).

The assemblage of a beneficial root endophyte can play an important role in assisting hosts as acquired immune systems, in supporting the plant immune system, and in defending against pathogens (seen in Figure 3). Specifically, upon the attack of pathogenic microorganisms, the interaction between plants and endophytes can enhance the expression of plant defense genes and help form papillae at the infiltration site of mycelium in order to defend against pathogens, thereby generating a pathogen immune system. The immune layer involves sensing MAMPs by PRRs and transmitting the perception of nonself signals to activate antimicrobial responses, limiting pathogen growth and initiating quantitative immune responses to control the host-microbial load [25,29]. Hence, plants can sculpt the root microbiome by stimulating immune signals to drive colonization of available microbial communities. To thwart microbial pathogens aboveground and plant viruses, plants turn on the induced systemic resistance (ISR), including defense-related phytohormones SA, JA, and gaseous ethylene, to mediate localized and systemic plant immune responses [62,63,64], thereby regulating root colonization by allowing potential nonpathogenic microorganisms to grow to improve the microbiome. Thus, ISR plays crucial roles in the signal network that regulates induced defense responses against biotic stresses. Additionally, endophytes can be beneficial by participating in essential metabolic pathways of plant tolerance to biotic stressors, including the ascorbate (AsA)-glutathione (GSH) cycle and the oxidative pentose phosphate pathway (OPPP). To defend against the attack of *Fusarium oxysporum* on cucumber roots, the endophyte *Trichoderma harzianum* can enhance the potential of antioxidant biosynthetic enzymes, reducing oxidative and nitrifying stress by reducing the transcription and accumulation of ROS and NO, respectively, which may help to improve redox balance, energy flow and defense reactions [65]. Moreover, beneficial endophytes can participate in detoxification and inhibit the gene encoding of harmful metabolites of pathogens. To defend pathogens in plant roots, central regulators of endophytic bacterial lifestyle, such as cyclic-di-GMP, are involved in the evasion of plants PTI immunity, with significant reduction in pathogen virulence during plant infection [66,67]. The latest research has demonstrated that genes encoding hydrolytic enzymes, which are involved in detoxification and redox homeostasis in *Serendipita vermifera*, are strongly induced, resulting in the induction of pathogen *Bipolaris sorokiniana* genes involved in secondary metabolism and a significant repression of genes encoding putative effectors upon confrontation with the endophytes [68].

Due to the abovementioned immune signals, the endosphere microbiome can manipulate plant-derived immune metabolites and different pathways to allow the colonization of potentially beneficial microorganisms that can induce systemic resistance without being blocked by the local root immune response. Therefore, the abundance of some root-colonizing endophyte families was increased at the expense of others in plants. Hence, maintaining endophyte-plant associations requires tightly regulated responses that elicit a host defense response, which mainly manifests in suppressing host defenses and endophytes’ excessive proliferation, as well as inhibiting endophyte-derived toxic proteins or metabolites.

## 5. Endophytic Fungi Are Critical for Host Health

**Figure 4:**
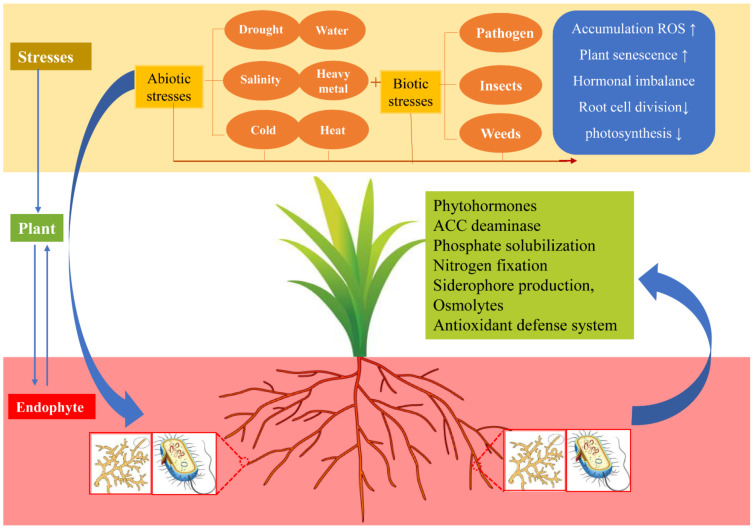
The plant–endophyte relationship between biotic and abiotic stress environments. Plants are subjected to a variety of biotic and abiotic stresses throughout their life, resulting in ROS accumulation, increased plant susceptibility, decreased photosynthesis, and decreased root differentiation, thus inhibiting growth and reproduction. In turn, the colonization of endophytes in plant tissues is conducive to the plant tolerance to a variety of stresses, which can promote plant growth and the bioavailability of nutrients, secrete antioxidants to reduce ROS damage, promote ACC deaminase to reduce ethylene decline, and produce siderophore to maintain iron homeostasis. Abbreviations: 1-aminocyclopropane-1-carboxylic acid (ACC), reactive oxygen species (ROS).

Hosting endophytes in plants are vital for the health of plants in their natural environment, so that they can cope with many stressors throughout their lifetime. As sessile organisms, plants are always under constant pressure and challenges. Microbial biopriming represents an adaptive strategy to improve the defensive capacity of plants that results in an increased resistance/stress tolerance, and/or a more aggravated defense response against the stress challenged conditions [69] (Figure 4). Recently, conscious agriculture, a serious alternative to conventional farming, has been proposed [70]. Well-understood microorganisms for plant biological control can support this. Characterizing and refining plant genotype-by-environment-by-microbiome-by-management interactions can accelerate the design and implementation of effective agricultural microbiome manipulation strategies for both consumers and producers of food supplies [71].

Plants can “cry for help” from their root endosphere when they are under attack by pathogens [72]. To adapt to adverse situations caused by biotic stress from bacteria, fungi, viruses, nematodes, and insects and enhance defense against a broad range of pathogens and insect herbivores, beneficial root-associated mutualists can sensitize the plant immune system by induced systemic resistance (ISR) [54]. Considering that managing species-rich communities of stress-associated endophytes remains a major challenge, core microbiomes have potential use in sustainable agroecosystems [73]. Root-associated mutualists, including *Trichoderma* and *mycorrhiza* species, sensitize the plant immune system for enhanced defense. Hub microorganisms are critical determinants of the microbiome interaction network structure [74]. With the help of endosymbiosis with AM fungi, potatoes can develop a defense against pathogen *Potato virus Y* [75]. Additionally, endophytes can manipulate epigenetic regulation in antagonistic bacterial-fungal interactions (BFI). Bioprospecting endophytes are capable of producing desired bioactive secondary metabolites, such as lipopeptide antibiotics, phenazine derivatives, and other antimicrobial metabolites, to directly inhibit pathogens [76]. Endophytic colonization of maize plants by *Metarhizium robertsii* can promote plant growth, alter defense gene expression in maize, and suppress the growth rate of black cutworm larvae [77].

Moreover, growth-promoting endophytes play a significant role in the alleviation of abiotic stress in plants (see in Table 1), enhancing host tolerance to adverse environments, such as drought, high salinity, cold, heat, and heavy metal stress [78]. Plant-endophyte partnership is a promising phytoremediation approach to remediate contaminated soil by organic pollutants [79], such as polycyclic aromatic hydrocarbon (PAHs) contaminants. The possible mechanism of abiotic and biotic tolerance in plants triggered by AMF includes phytohormones regulation, exopolysaccharides production, phosphate solubilization, nitrogen fixation, siderophore production, accumulation of various osmolytes (such as proline, sugars, amino acids, polyamines and betaines), alteration of antioxidant defense system, and induction of stress-responsive genes in plants [80,81,82,83]. In particular, in addition to the direct absorption of phosphate by root epidermal cells, AM symbiotic plants can continuously respond to low nitrogen and phosphorus concentrations through specific mycorrhizal phosphate absorption pathways and increase water supply under stress conditions [83]. Dual inoculation of mycorrhizae fungi seems to be an effective strategy for improving plant growth under stress conditions, compared to that of individual inoculation [84]. Notably, root endophytes drive direct integration of abiotic stress and immunity. In axenic Pi conditions, the transcriptional regulator *PHR1* in endophytes can directly integrate plant immune system output and plants’ adaptive phosphate starvation responses (PSR) [85]. Moreover, iron homeostasis plays an important role in plant immunity. Beneficial microbes within the rhizosphere antagonize soil-borne pathogens through siderophore-mediated competition for iron [86]. Thus, endophytes play an important role in resisting abiotic and biotic stresses for plants.

### 5.1. Fungi Change ROS Hubs in Plant Responses to Stresses

In nature, plants are subject to a variety of biotic and abiotic stress signals’ cross-talk. Phytohormones, including auxins, gibberellins (GA), cytokinins (CK), abscisic acid (ABA), ET, SA, JA, brassinosteroids (BR), and strigolactones, play critical roles in helping plants to adapt to adverse abiotic and biotic stresses and in discriminating the intricate web of cross-talk [101,102]. Notably, ROS is one of the most common plant responses to abiotic and biotic stresses, representing a point at which various signaling, composed of pathways upstream and downstream of signaling components, come together, such as calcium, redox homeostasis, membranes, G-proteins, MAPKs, plant hormones including SA, JA, ABA, and ethylene, as well as transcription factors [103,104]. Enhanced metabolite flux through the photorespiratory pathway, caused by various abiotic stresses, leads to the overproduction of ROS in plants. ROS is highly reactive and toxic and cause damage to proteins, lipids, carbohydrates, and DNA, ultimately resulting in oxidative stress [105]. The endophyte-mediated plant antioxidant system plays an important role in promoting plant response to the above abiotic stresses and alleviating stress damage through controlling ROS damage. Under Cd stress, colonizing dark septate endophytes of maize results in a marked tolerance to Cd via affecting physiological, cytological, and genic aspects. The process is related to a significant decrease in Cd phytotoxicity and a significant increase in maize growth by triggering antioxidant systems, altering metal chemical forms into inactive Cd, and repartitioning subcellular Cd into the cell wall by changing the transcript levels of key genes involved in Cd transport and detoxification [106]. The water stress tolerance of *Nicotiana benthamiana* inoculated with fungi is associated with the increased activity of antioxidant enzymes (including catalase, peroxidase, and polyphenol oxidase), decreased ROS production, and decreased electrical conductivity. In addition, several genes previously identified as drought-induced are significantly upregulated [107].

### 5.2. Plants Genotype and Metabolic Signals to Recruit Favorable Microbes

Notably, a plant’s genotype influences its microbiome composition, shaping their microbiome to enhance defense and mitigate the trade-off between growth and defense against pathogens [108]. During microbial infection, host RNAi machinery is highly regulated and contributes to reprogramming gene expression and balancing plant immunity and growth [109]. miRNAs have always been demonstrated to be implicated in the disease resistance of host plants by inducing cross-kingdom gene silencing in pathogenic fungi [110,111]. However, during AM symbiosis, plant cells undergo a complex reprogramming, resulting in profound miRNA changes in response, to recruit beneficial endophytes. miRNAs could be an important part of the regulatory network leading to symbiosis development [112,113]. miR171b derived from plants stimulates AM symbiosis and protects the target gene *LOM1* from negative regulation by other miR171 family members [113]. Conversely, upon abiotic stresses, plants can secrete exosome-like EVs to deliver targeted sRNAs into pathogens and pests to silence virulence genes and inhibit their virulence [114]. Hence, in some cases, miRNAs in plants react in opposite ways to beneficial endophytic microbes and pathogens [115], suggesting that plants can help endophytes escape their immune signals. However, the reason for this difference has not been fully revealed. This may be due to the endogenous silencing small RNAs in plants [116]. Additionally, long noncoding RNAs (lncRNAs) in plants have a regulatory network responsive to AMF colonization in maize roots, involved in the regulation of bidirectional nutrient exchange between plant and AMF as mimicry of microRNA targets [117]. The symbiosis-related regulatory networks of differentially expressed lncRNAs-mRNAs-miRNAs were also constructed. Nevertheless, genes associated with endosymbiosis signaling are invariantly conserved in all land plant species possessing intracellular symbionts. Common symbiosis signaling pathways co-evolved with intracellular endosymbioses, from the ancestral AM to recent ericoid and orchid mycorrhizae in angiosperms and ericoid-like associations of bryophytes [42]. Additionally, endophyte symbiotic microbes can induce major transcriptional changes in plants, such as the sugar transporter SWEET for sucrose secretion out of cortical cells, ultimately reprograming the whole plant carbon partitioning [118]. For beneficial endophyte growth and development, plants can transfer a lipid cross-kingdom to its endophytic AMF, which lacks genes encoding fatty acid synthase I subunits [119]. Moreover, together with genotype, plant age and domestication may influence these fungal communities [120]. Some legumes, such as members of the invert repeat lacking clade, produce up to several hundred antimicrobial peptides to control bacteroid cell division and development [121]. Plant-associated fungal communities are more strongly influenced by host genetic factors and plant breeding than by bacterial communities [122].

Apart from producing antimicrobial compounds to cope with pathogens, plant root exudates, which are composed of low-molecular-weight compounds such as sugars, amino acids, organic acids, and phenolics, and high-molecular-weight compounds, have also acted as a source of molecular signals to actively recruit members of the soil microbial community for positive feedback [123]. Endosymbiosis-associated compounds, such as flavonoides and nonflavonoid molecules derived from plants [124], can attract rhizobia to roots and activate the genes responsible for activation of nod genes, along with branching factors exuded by plants for AMF [125]. Plastid proteins secreted by plants are also indispensable for microbial admission into plant cells and act upstream of intracellular Ca^2+^ spiking.

Moreover, plants secrete a variety of chemicals to attract beneficial microbes and defend against pathogens during endophytic interactions [126]. Plant defensive secondary metabolites released from the roots of grain can be used as chemical attractants or inhibitors to change root-related fungal and bacterial communities, inhibit herbivore performance in next-generation plants, or attract endophytic bacteria in roots to colonize [37,127]. Regulating the synthesis of plant metabolites for the growth of endophytes is a strategy for plants to cope with stress. Tryptophan-derived secondary metabolites in Brassicaceae plants regulate beneficial interactions with fungal endophytes under nutrient-limiting conditions. In addition, the nutritional status of endophytes based on host carbohydrate dynamics is another strategy to establish a symbiotic relationship under pressure. Under nitrogen-poor conditions, the presence of the fungal endophyte *Diaporthe* *liquidambaris* mediates host carbohydrate dynamics, including promoting chlorophyll biosynthesis and water-soluble carbohydrate accumulation, and induces an enhanced mutualistic system [128]. Hence, endophyte can be utilized as a biotic resource that effectively minimizes damage towards plants from environmental stresses. The application of microbiome-based agro-management practices and improved plant lines could lead to a better use of plant endophytes. Stress-tolerant endophytic fungi improve plant survival by up to 4 d in low water and up to 2 d in high water, relative to the control plants. Additionally, fungi from wetter and cooler sites are less beneficial, with 8–24% lower plant biomass and up to 450% greater water loss, compared with plants inoculated with fungi from warmer, drier sites [129].

### 5.3. Endophytic Fungi Can Affect the Growth and Differentiation of Plant Roots

Apart from reshaping the endosphere microbe community and enhancing pathogen defense, the plant-endophyte relation can enhance plant root growth [130]. Beneficial endophytic fungi can synthesize and release phytohormones, such as auxins, cytokinins (CKs), gibberellins (GAs), and ethylene (ET), along with auxin indole-3-acetic acid (IAA) which are able to regulate multiple physiological processes of root initiation, elongation, and root hair formation [16]. By changing the above plant endogenous signaling pathways, as well as quorum sensing auto-inducers of N-acyl homoserine lactones (AHL) [131], plant growth-promoting beneficial microbes can affect the division and differentiation of plant cells, leading to changes in the architecture of root system, which contributes to enhance the growth of the plant shoot. Independent of the above pathways, the balance of ROS change, which is affected by endophytes, also has a great influence on plant root differentiation [132]. Moreover, an arsenal of apoplast proteins secreted by endophytic fungi can facilitate inter and intracellular colonization of plant tissues [48]. During the accommodation of symbiotic fungus, AMs hosts can activate cell-division-related mechanisms by upregulating endocytic effectors *TPLATE*, *KNOLLE*, and Cyclinlike 1 (*CYC1*) in colonized cells of root cortex, alongside activating endocytic markers adaptor-related protein complex 2 alpha 1 subunit (*AP2A1*) and clathrin heavy chain 2 (*CHC2*) during cell plate formation [133].

### 5.4. The Mutual Beneficial Relationship Is Not Always Durable and Reliable

However, not all endophytes can establish a lasting beneficial symbiotic relationship within plants; ecological and host factors are important in shaping microbial hub taxa variation in plants [74]. Interestingly, individual endophytes are phenotypically plastic, and can easily switch between an endophytic and necrotrophic lifestyle. According to a maximum-likelihood analysis combined with ancestral character mapping by maximum parsimony, it has been revealed that some fungal lineages among 163 fungi strains had switched multiple times between a necrotrophic and an endophytic lifestyle [134]. Sometimes, plants are willing to take risks under nutrient starvation to facilitate associative microorganism colonization and capture nutrients [135]. Stress can activate MAPK signaling, which is required to maintain a fungal endophyte-grass symbiosis. The formation of ROS by the fungal NADPH oxidase (Nox) complex is essential for the maintenance of a symbiotic interaction [136]. Notably, the disruption of fungal MAPK signaling or Nox complex leads to a breakdown in the symbiosis [136,137]. When plants are infected by endophytic fungus, which is a lack of functional Nox complexes or stress-activated MAPK signaling, the host can exhibit a stunted phenotype and premature senescence, while the fungus shifts from restricted growth to proliferative growth [137]. Notably, not all endophytes show inhibition of host growth under phosphorus-deficient conditions. It could also be a nutrient status that might facilitate the transition from pathogenic to beneficial lifestyles. It has been demonstrated that the host’s phosphate starvation response (PSR) system can control *Colletotrichum tofieldiae* root colonization and is needed for plant growth promotion (PGP). PGP also requires PEN2-dependent indole glucosinolate metabolism, which is a component of innate immune responses for restricting *Colletotrichum tofieldiae* colonization, indicating a functional link between innate immunity and the PSR system during beneficial interactions with *C. tofieldiae* [138]. On the other hand, the master transcriptional regulator PHR1 of PSR directly activates endophytic microbiome-enhanced responses to phosphate limitation while repressing microbially driven plant immune system outputs [85]. Thus, defense responses are activated under phosphate-sufficient conditions. Therefore, the effect of endophytes on plants under low phosphorus is controversial, and requires further research. The specific reason may be that different endophytes have different effects on plants in the case of nutrient deficiency. Additionally, it indicates that, when plants have pathological changes caused by endophytes in the nutrient-deficient soil, the counterpart endophyte can be used as a biological enhancer to improve plant health.

Moreover, the accumulating evidence reveals that fungal offspring, including sexual or asexual fungus spore, does not guarantee mutualism or stability of the interaction with the host. Gene-deleted mutants of Δ*sakA*, heterochromatin 1 protein (*HepA*), switch the fungal interaction with the host from mutualistic to pathogenic [139,140], which is due to disruption of signaling in plant-microbiome endosymbiosis. Additionally, endophytic fungi, which have a biological control function and a health-promoting effect on host plants, could be the pathogen of other plants. *Pantoea ananatis*, which promotes growth of pepper, cucumber, tomato, melon, and rice plants [141], may act as a virulence regulator to some types of plants, owing to its Hfq-dependent sRNAs [142,143]. Likewise, the fungus *Verticillium dahliae*, which causes wilts of several hundred plant species, such as potato and mint, colonizes asymptomatic hosts, such as mustards and barley, as endophytes [144]. Hence, not all endophytes can form a firmly beneficial symbiotic relationship within all hosts; this depends on plant type, endophyte genotype, and physiological environment. Notably, the definition of a class of microbe as pathogen or endophytes should take into account interspecific host differences.

## 6. Conclusions and Future Perspectives

To conclude, as a beneficial companion in plants, endophytes help plants to resist both biotic and abiotic stresses by regulating the immune response of plant-microbe interactions and stimulating the production of metabolites. Therefore, in general, endophytes have a broad application prospect for use as biological control agents and biological fortifiers. However, there is still a long way to go to integrate endophytes into conscious agricultural production.

Firstly, not all endophytes are inherently useful, which is strongly dependent on plant species and genotypes. In addition, stress can also largely reshape the endophyte community. Therefore, a specific plant-endophytic stress model system needs to be well developed. In turn, the model can also be used to predict and design synthetic endophytic communities for predictable plant phenotypes and alter plant response to various stresses. However, as far as current knowledge is concerned, due to the high genetic variability and functional diversity of microorganisms, plant growth cannot be predicted by certain phylogeny and species identity (such as AMF), which requires more multiple sites data [145]. Secondly, there are many endophytes in plants, but not all endophytes are beneficial. Therefore, it is difficult to identify the endobiont group by a knowledge-driven selection of endophyte. Hence, determining the core microbe and metagenome of endophytes in the corresponding model is of great importance. Thirdly, in order to synthesize plant active products by endophyte, it is necessary to understand the relationship between endophyte genes and plant bioactive compounds. Consequently, microbial cells from endophytes related to plant metabolites can be green factories for the advanced production of plant secondary metabolites. Therefore, the model of a plant’s active metabolite-endophyte-synthetic gene can be established for the large-scale synthesis of medicinal metabolites. However, the presence of numerous uncharacterized biosynthetic genes in plant and endophyte genomes suggests that many molecules related to biosynthesis of active ingredients by plant-endophyte interactions remain unknown. Hence, no study has yet claimed the cost-effective yield of bioactive metabolites from fungal endophytes.

Notably, the revolutionary techniques of the CRISPR/Cas9 system can promise to serve as an ideal platform to know the basics of plant-microbe interactions in a fast-forward way by developing plants/microbes relevant for agricultural application, and conducting functional studies of biotic and abiotic stress-related genes in plant-microbe interactions. Although it has been demonstrated that the CRISPR/Cas9 system could greatly facilitate functional analyses of endophyte-related genes in endosymbiotic plants [146,147], nevertheless, the technique is still too much in its infancy to investigate plant-microbe interactions. Consequently, identifying plenty of individual plant or microbial candidate genes governing agronomic traits will facilitate CRISPR-based applications in sustainable agricultural practice under community-level molecular mechanisms and biosynthetic pathways of novel natural bioactive compounds. Integrated with multi-omics technologies, CRISPR/Cas9 technology will bring landmark achievements to endophyte-centered ecological agriculture.

## Figures and Tables

**Table 1:** Endophytic fungi promote plant tolerance to both biotic and abiotic stresses.

Endophytic Fungi Derived from Plants	Endophytic Fungi	Host Plant	Stress Type	Mechanism of Action	Reference
/	*Paecilomyces formosus* LHL10, *Penicillium funiculosu-m* LHL06	Soybean (*Glycine max* L.)	Heavy metals; drought, high temperature	Promote photosynthetic activity, glutathione, catalase, and SOD activities; decrease lipid peroxidation; downregulate heavy metal ATPase gene expression; reduce ABA and IA levels	[87]
*Suaeda salsa*	*Sordariomycetes* sp.	*Oryza sativa*	Heavy metals: Pb^2+^	Maintain photosystem II function	[88]
Tomato	*Penicillium janthinellum* LK5	tomato (*Solanum lycopersicum*)	Heavy metals: Al	Reduce damage to root structure and essential lipid membrane Regulate antioxidants and endogenous salicylic acid	[89]
*Aeschynomene fluminensis*, Polygonum acuminatum	Aspergillus sp. A31, Curvularia geniculata P1, Lindgomycetaceae P87, Westerdykella sp. P71	*Aeschynomene fluminensis*, *Zea mays*	Heavy metals: Hg	IAA production; phosphate solubilization; siderophore production; decrease mercury translocation factor; remediate mercury in vitro via mycelial volatilization and biosorption/bioaccumulation	[90]
Cucumber	*Paecilomyces formosus* LHL10	*Glycine max* L.	Heavy metals: Ni	Enhance chlorophyll content; Reduce lipid peroxidation; Antioxidant production (LNA, GSH, PPO, CAT, SOD) Enhance the translocation of Ni from the root to the shoot	[91]
Cucumber	*Penicillium funiculosum* LHL06	*Glycine max* L.	Heavy metals: Ni, Cu, Pb, Cr, Al)	GA production; IAA production; downregulation of heavy metal transporter genes; activate signaling network of stress-responsive hormones and antioxidant systems	[92]
*Bischofia* *polycarpa*	*Phomopsis liquidambaris* B3	Rice (*Oryza sativa* L.)	Organic pollutants	Increase root viability, chlorophyll content and energy supply; increase the PPO activity and SOD activity in shoot; degrade PHE absorbed into rice;	[93]
*Clerodendrum serratum* (L) Moon	*Streptomyces sp*. GMKU 336	Rice	Salinity stress	ACCD production; removal of active oxygen; counter ion content	[94]
/	*Piriformosporaindica*	*Arabidopis thaliana*	Salinity stress	Increase expression of the ion channels; increase plant biomass, lateral roots density, and chlorophyll content	[95]
/	*Arbuscular mycorrhizal fungi*	Ephedra foliata Boiss	Drought	Upregulate antioxidant defense system; synthesis of osmolytes; maintain phytohormone levels;	[96]
*Clerodendrum serratum* (L.) Moon	*Streptomyces sp*. GMKU 336	Mung bean	Water	ACCD production; enhance chlorophyll content and biomass;	[97]
Potato	*Rhizophagus irregularis*	Potato	Biotic stress (potato virus Y)	Decrease the level of shoot- and root-derived H_2_O_2_; mask infection by PVY	[98]
/	*Trichoderma harzianum* T-78	Tomato (*Solanum lycopersicum*)	Biotic stress (*Meloidogyne incognita*)	Prime SA-regulated defences; enhanced JA-regulated defences;	[99]
*Panax notoginsen-g*	*Trichoderma gamsii* YIM PH30019	*Panax notoginseng*	Biotic stress (Pathogen)	Produce effective antagonistic active ingredients (dimethyl disulfide, dibenzofuran, methanethiol, ketones)	[100]
